# Columnar structured FePt films epitaxially grown on large lattice mismatched intermediate layer

**DOI:** 10.1038/srep34637

**Published:** 2016-09-30

**Authors:** K. F. Dong, J. Y. Deng, Y. G. Peng, G. Ju, G. M. Chow, J. S. Chen

**Affiliations:** 1 School of Automation, China University of Geosciences, Wuhan 430074, China; 2 Department of Materials Science and Engineering, National University of Singapore, Singapore 117576, Singapore; 3 Seagate Technology, Fremont, CA 94538, USA

## Abstract

The microstructure and magnetic properties of the FePt films grown on large mismatched ZrN (15.7%) intermediate layer were investigated. With using ZrN intermediate layer, FePt 10 nm films exhibited (001) texture except for some weaker FePt (110) texture. Good epitaxial relationships of FePt (001) <100>//ZrN (001) <100>//TiN (001) <100> among FePt and ZrN/TiN were revealed from the transmission electron microscopy (TEM) results. As compared with TiN intermediate layer, although FePt-SiO_2_-C films grown on ZrN/TiN intermediate layer showed isotropic magnetic properties, the large interfacial energy and lattice mismatch between FePt and ZrN would lead to form columnar structural FePt films with smaller grain size and improved isolation. By doping ZrN into the TiN layer, solid solution of ZrTiN was formed and the lattice constant is increased comparing with TiN and decreased comparing with ZrN. Moreover, FePt-SiO_2_-C films grown on TiN 2 nm-20 vol.% ZrN/TiN 3 nm intermediate layer showed an improved perpendicular magnetic anisotropy. Simultaneously, columnar structure with smaller grain size retained.

The areal density of hard disk drives needs to be increased to meet the huge demand of storing exponentially increasing digital information. FePt based heat assisted magnetic recording (HAMR) media has drawn a lot of attention due to its ability to extend the areal density up to 5 Tb/in^2^ 1,2. For application of FePt thin film in HAMR, the FePt film should have a good (001) texture, large magnetocrystalline anisotropy, small grain size with a narrow size distribution and well isolated columnar structure. (001) textured FePt films with large magnetocrystalline anisotropy and small grain size have been reported by epitaxial growth on single crystal substrate or polycrystalline underlayers^3^,^4^,^5^,^6^,^7^,^8^,^9^,^10^,^11^,^12^,^13^,^14^,^15^. Till now, the lattice mismatch of substrates used to induce FePt (001) textured films are only from −1.63% to 9.1%, such as 9.1% for TiN^8^,^9^, 8.63% for MgO^3^,^4^,^5^,^6^,^7^, 4.6% for MgAl_2_O_4_^10^, 3.44% for KTaO_3_^15^, 1.44% for SrTiO_3_^10^,^11^,^12^,^13^,^14^,^15^ and −1.63% for LaAlO_3_^12^,^15^, respectively. It is found the lattice mismatch plays a crucial role in the growth of epitaxial thin films^16^,^17^. Y. Chen^18^ *et al.* had proposed that the mismatched film preferred island structure when the size of the mono epilayer exceeded the critical size, which is inversely proportional to the mismatch strain *ε*_*xx*_^6^. Therefore, larger lattice mismatch (larger than TiN) will be favor for the FePt films with smaller grain size. However, the large mismatch may not be favored for good (001) texture and perpendicular anisotropy. Until now, the effects of larger lattice mismatch (11–15%) on the magnetic properties and microstructure of FePt films have not been reported and the epitaxial growth correlation between FePt and larger mismatched intermediate layer still remains unclear. In this paper, we deposited FePt thin films on ZrN intermediate layer to systematically investigate the larger lattice mismatch effects on the microstructure and magnetic properties of FePt films, and the lattice mismatch between FePt films and ZrN layer is 15.7%.

## Results

**Figure 1:**
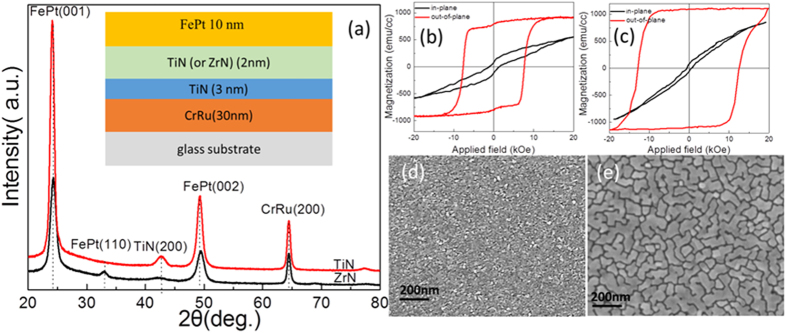
(**a**) XRD 2*θ* spectra, (**b**,**c**) M-H loops and (**d**,**e**) SEM images of FePt 10 nm films grown on different intermediate layer, (**b**) and (**d**) for ZrN 2 nm/TiN 3 nm intermediate layer, (**c**) and (**e**) for TiN 5 nm intermediate layer. The inset is the film structure.

Figure 1(a) shows XRD 2*θ* spectra of FePt 10 nm films grown on different intermediate layer. It can be seen that the FePt films grown on TiN intermediate layer exhibited (001) preferred orientation. This is due to the epitaxial growth of the FePt on the (200) textured TiN intermediate layer^19^,^20^. For FePt films grown on ZrN intermediate layer, in addition to the FePt (001) and (002) peaks, FePt (110) peaks were also observed. As compared with the intensities of FePt (001) and (002) peaks, the intensity of FePt (110) peak was much weaker, indicating the (001) texture was dominant in this FePt film. However, No peaks ZrN were shown in Fig. 1(a), which was due to the small quantity of ZrN. Although both FePt films revealed perpendicular anisotropy, the FePt films grown on ZrN/TiN intermediate layer (Fig. 1b) showed a bigger open-up in in-plane loops than that of using TiN intermediate layer (Fig. 1c) due to the formation of some (110) phase. All these indicated FePt films grown on TiN intermediate layer had better (001) texture and perpendicular anisotropy. Moreover, the saturated magnetization (1100 emu/cc) of FePt 10 nm films grown on TiN intermediate layer was larger than that (914 emu/cc) of FePt films grown on ZrN intermediate layer, which may be caused by the interlayer diffusion between the FePt and ZrN. The SEM images of the FePt films are shown in Fig. 1(d,e). As seen, island growth of FePt grains with distinct grain boundary was formed for both intermediate layers. Moreover, the grain size of FePt grown on ZrN/TiN was smaller than that of grown on TiN intermediate layer.

**Figure 2:**
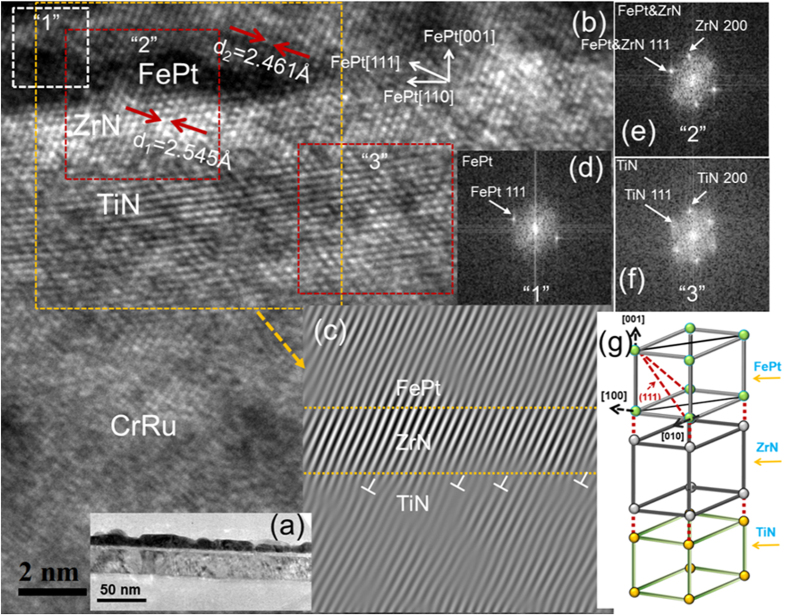
(**a**) Low magnifications cross sectional TEM images and (**b**) high resolution cross sectional TEM images of FePt 10 nm film on ZrN intermediate layer, (**c**) the selected area inverse fast Fourier transform, (**d**–**f**) are the corresponding selected area electron diffraction (SAED) patterns of FePt, FePt&ZrN and TiN, as well as (**g**) the schematic drawing of FePt grown on ZrN/TiN intermediate layer. The dislocations are marked with “⊥”.

The cross section TEM results of FePt 10 nm grown on TiN intermediate layer had been reported previously (no shown here) and good epitaxial relationship of FePt (001) <100>//TiN (001) <100> had been confirmed^20^. The in-plane mismatch *ε* *=* *(a*_*sub*_ *− a*_*FePt*_*)/a*_*sub*_between TiN and FePt was around 9.1%. In order to investigate the epitaxial growth relationship between FePt and ZrN, the cross-sectional TEM were carried out. Figure 2 illustrates (a) low magnifications cross sectional TEM images and (b) high resolution cross sectional TEM images of FePt 10 nm film on ZrN intermediate layer, (c) the selected area inverse fast Fourier transform, (d), (e) and (f) are the corresponding selected area electron diffraction (SAED) patterns of FePt, FePt&ZrN and TiN. It can be seen from Fig. 2(a) that FePt films with island growth structure were formed on ZrN/TiN intermediate layer. Crystallized ZrN was observed with lattice plane spacing of 2.545 Å (Fig. 2b), which represented ZrN (111) lattice planes. In FePt layer, FePt (111) lattice planes with lattice plane spacing of 2.461 Å were also exhibited, much larger than the bulk value (2.192 Å), which was caused by the tensile strain between FePt and ZrN. The interfaces between FePt and ZrN were sharp and clear. In the IFFT images (Fig. 2c), the (111) lattice planes of FePt and the (111) lattice planes of ZrN were connected together, and no dislocations were formed in the selected area, implying a good epitaxial growth of FePt on the ZrN intermediate layer. In addition, some dislocations were formed at the interface of TiN and ZrN, which was due to the larger lattice mismatch between ZrN and TiN. The dislocations were marked in Fig. 2(c) with “⊥”. As seen from the SAED patterns, the FePt (111) and the ZrN (111) spots overlapped (Fig. 2e), indicating the axis of FePt aligned very well along the ZrN (111) axis. It can be confirmed the epitaxial relationship of FePt (001) <100>//ZrN (001) <100>//TiN (001) <100>. The schematic drawing of the FePt unit cells arranged on the ZrN/TiN intermediate layer are also shown in Fig. 2 (g). However, the appearance of (110) peaks indicated the existence of some grains with epitaxial relationship different from (001)[100]//(001)[100], regretfully which were not observed in our TEM measurement due to the small quantity of FePt (110) phase and the narrow TEM observation area.

## Discussion

**Figure 3:**
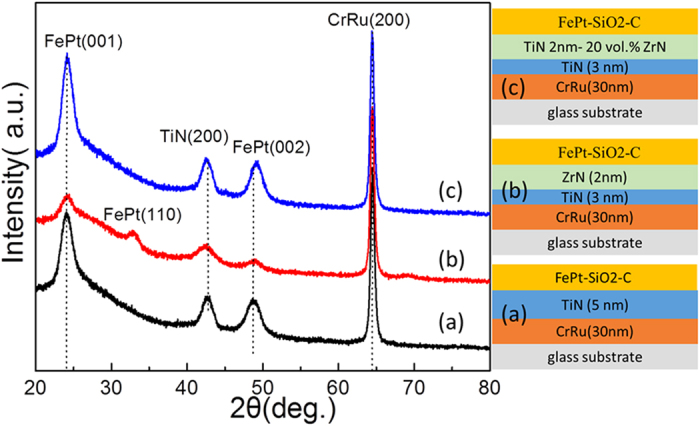
XRD 2*θ* spectra of FePt 4 nm-SiO_2_ 35 vol.%-C 20 vol.% films with different intermediate layers, (**a**) TiN 5 nm intermediate layer, (**b**) ZrN 2 nm/TiN 3 nm intermediate layer and (**c**) TiN 2 nm-20 vol.% ZrN/TiN 3 nm intermediate layer. The insets are the corresponding film structures.

Based on the results above, in order to further study the effect of large lattice mismatch on microstructure and magnetic properties of FePt films, FePt 4 nm-SiO_2_ 35 vol.%-C 20 vol.% films were fabricated on these intermediate layer. Figure 3 shows the the XRD θ–2θ scans of FePt 4 nm-SiO_2_ 35 vol.%-C 20 vol.% films with different intermediate layers, (a) TiN 5 nm intermediate layer, (b) ZrN 2 nm/TiN 3 nm intermediate layer and (c) TiN 2 nm-20 vol.% ZrN/TiN 3 nm intermediate layer. FePt-SiO_2_-C film showed very good (001) texture with using TiN intermediate layer (Fig. 3a). With using ZrN 2 nm/TiN 3 nm intermediate layer, FePt (001) and (002) peaks became weak and FePt (110) peak appeared, indicating some in-plane variance was formed (Fig. 3b). With introducing 20 vol.% ZrN into TiN immediate layer (Fig. 3c), the TiN (200) peak position shifted to lower angle, suggesting the lattice constant of TiN increased. As calculated by the peak position of TiN (200), the lattice constant of TiN increased from 4.247 Å to 4.258 Å, which is due to the formation of solid solution between TiN and ZrN (4.57 Å)^21^. The in-plane mismatch between TiN 2 nm-20 vol.% ZrN/TiN 3 nm intermediate layer was 0.2%. Moreover, the FePt (002) peak position shifted to higher angle and the value of I_(001)_/I_(002)_ increased from 2.45 to 3.03. This indicates ZrN doped into the TiN intermediate layer could cause the improvement of FePt (001) texture and the enhancement of chemical ordering. As compared with the FePt 10 nm films with using TiN 5 nm intermediate layer and ZrN 2 nm/TiN 3 nm intermediate layer, the FePt-SiO_2_-C films with the same intermediate layer can obtain smaller grain size with better grain isolation.

**Figure 4:**
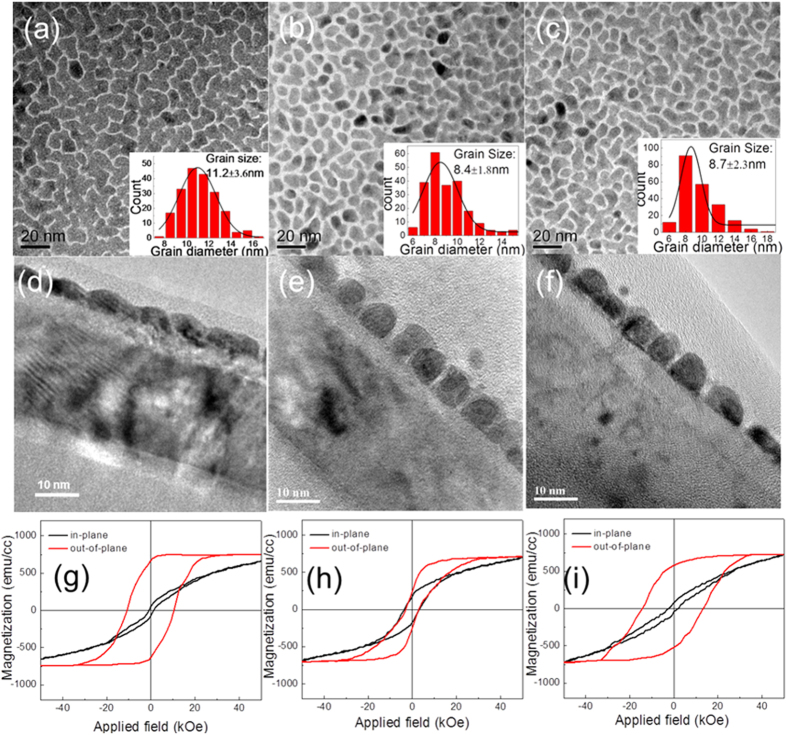
(**a**–**c**) Planar view TEM images, (**d**–**f**) low magnifications cross sectional TEM images and (**g**–**i**) the corresponding M-H loops of FePt 4 nm-SiO_2_ 35 vol.%-C 20 vol.% films with different intermediate layers, (**a**,**d**,**g**) for TiN 5 nm intermediate layer, (**b**,**e**,**h**) for ZrN 2 nm/TiN 3 nm intermediate layer, and (**c**,**f**,**i**) for TiN 2 nm-ZrN 20 vol.%/TiN 3 nm intermediate layer. The insets are the corresponding grain size distribution.

Figure 4 illustrates the microstructure and magnetic properties of FePt 4 nm-SiO_2_ 35 vol.%-C 20 vol.% films with different intermediate layers. As seen from the planar view TEM images (Fig. 4a), the grain shape of FePt films grown on TiN intermediate layer was maze-like. With using ZrN/TiN intermediate layer, the grain boundaries became more distinct, and the grain changed from maze-like to equiaxed shape (Fig. 4b). Furthermore, the grain size was reduced from 11.2 ± 3.6 nm to 8.4 ± 1.8 nm with an improved grain size distribution, which was consistent with the assumption above- larger lattice mismatch (larger than TiN) will be favor for the FePt films with smaller grain size. The corresponding low- magnifications cross sectional TEM images (Fig. 4d,e) exhibited FePt grains with one layer structure were formed on both TiN and ZrN/TiN intermediate layer. More importantly, well isolated FePt grains with columnar structure were obtained on ZrN/TiN intermediate layer. However, FePt-SiO_2_-C film grown on ZrN/TiN intermediate layer showed isotropy magnetic properties with out-of-plane coercivity of 3.1kOe and in-plane coercivity of 3.6 kOe (Fig. 4h). Considered FePt films grown on TiN can obtain good perpendicular magnetic anisotropy (Fig. 4g) and TiN and ZrN are complete solution^21^, ZrTiN intermediate layer was proposed to improve the magnetic properties and maintain the columnar structure of FePt. As compared with results of FePt films grown on TiN intermediate layer, FePt-SiO_2_-C film grown on TiN 2 nm-20 vol.% ZrN/TiN 3 nm intermediate layer shows smaller grain size of 8.7 ± 1.8 nm (Fig. 4c) and well isolated one layer columnar structure (Fig. 4f). Moreover, the perpendicular magnetic anisotropy was improved. The perpendicular coercivity H_c⊥_increased from 10.6 kOe with TiN intermediate layer to 14.7 kOe with TiN 2 nm-20 vol.% ZrN/TiN 3 nm intermediate layer (Fig. 4i). The saturated magnetization *M*_*s*_ of all samples were around 750 emu/cc and the *M*_*s*_ of FePt film was a little larger than that of FePt films grown on ZrN intermediate layer.

**Figure 5:**
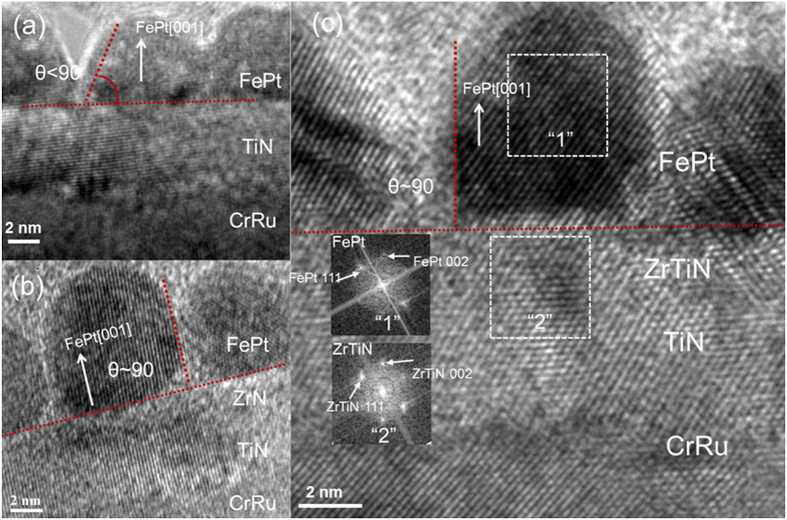
High resolution cross sectional TEM images of FePt 4 nm-SiO_2_ 35 vol.%-C 20 vol.% films with different intermediate layers, (**a**) TiN 5 nm intermediate layer, (**b**) ZrN 2 nm/TiN 3 nm intermediate layer and (**c**) TiN 2 nm-ZrN 20 vol.%/TiN 3 nm intermediate layer. The insets in (**c**) are the corresponding selected area electron diffraction (SAED) patterns of FePt and ZrTiN.

In order to further investigate the microstructure evolution of FePt with different intermediate layers, high resolution TEM was carried out. The results are shown in Fig. 5. FePt films grown on a pure TiN intermediate layer exhibited semispherical grains with a contact angle smaller than 90° (Fig. 5a), whereas the film grown on ZrN/TiN showed rectangular grains with a contact angle of around 90° (Fig. 5b). According to Young’s equation, 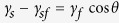 (where *γ*_*s,*_ *γ*_*f*_ and *γ*_*fs*_ are the surface energy of the substrate, the surface energy of the films, and the interfacial energy between the substrate and the films, respectively), *θ* is the contact angle, a large contact angle corresponded to the small surface energy of the substrate and the large interfacial energy. The surface energy of TiN and ZrN are *γ*_*TiN*_ ≈ 1.28 J/m^2^ and *γ*_*ZrN*_ ≈ 1.44 J/m^2^ 22, respectively. As *γ*_*ZrN*_ is larger than *γ*_*TiN*_, thus the large contact angle obtained by using ZrN intermediate layer was caused by the large interfacial energy between FePt and ZrN, which would promote island growth and thus good grain isolation. Although the larger surface energy of ZrN was not benefit for island growth of FePt films, the smaller grain size with good grain isolation and equiaxed shape of FePt-SiO_2_-C films with ZrN intermediate layer was due to the larger lattice mismatch and large interfacial energy between FePt and ZrN. On the other side, the small interfacial energy between FePt and TiN would cause to form maze-like FePt grains.

**Figure 6:**
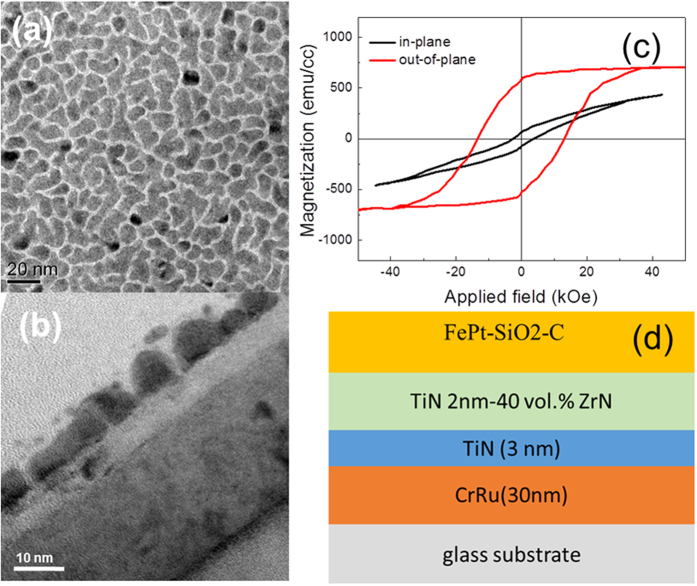
(**a**) Planar view TEM images, (**b**) low magnifications cross sectional TEM images, (**c**) the corresponding M-H loops and (**d**) the film structure of FePt 4 nm-SiO_2_ 35 vol.%-C 20 vol.% films on TiN 2 nm-ZrN 40 vol.%/TiN 3 nm intermediate layer.

Figure 5(c) revealed the FePt grains with (001) orientation were epitaxially grown on the (200) textured ZrTiN intermediate layer and the atomic planes across the ZrTiN and FePt interface matched well with each other. The contact angle was around 90°. It appears that ZrTiN intermediate layer is continuous with good crystallinity and clear ZrTiN/FePt interface. More importantly, although 20 vol.% ZrN was doped into TiN, no phase separation is observed in ZrTiN intermediate layer. Therefore, the ZrTiN intermediate layer is a solid solution of f.c.c TiN and f.c.c ZrN. As can be seen from the SAED patterns in Fig. 5(c), (001) and (111) axis of *L*1_0_ FePt aligned very well along ZrTiN (002) and (111) axis, respectively, confirming the epitaxial relationship of FePt (001) <100>//ZrTiN (001) <200>. These results confirmed that doping 20 vol.% ZrN into the TiN layer would increase the interfacial energy and the lattice constant, which would be benefit to get the large contact angle and promote island growth and thus obtain good grain isolation. Simultaneously, the optimal lattice constant with 20 vol.% ZrN doping would maintain good epitaxial growth between FePt and ZrTiN and do not deteriorate the perpendicular anisotropy. More than that, the improved isolation of FePt grains would enhance the perpendicular anisotropy of FePt films and make the magnetic properties better than that of using TiN intermediate layer (Fig. 4i). Further increase in the ZrN doping concentration to 40 vol.% caused the deterioration of the isolation and the perpendicular anisotropy (perpendicular coercivity H_c⊥_ ≈ 13.5 kOe) of FePt-SiO_2_-C films as shown in Fig. 6.

## Methods

### FePt films fabrication

FePt 10 nm and FePt 4 nm-SiO_2_ 35 vol.%-C 20 vol.% films were all fabricated on three kinds of intermediate layers by a magnetron sputtering system with a base pressure better than 2 × 10^−8^ Torr. One intermediate layer is TiN (5 nm)/CrRu (30 nm)/glass. The other intermediate layer is ZrN (2 nm)/TiN (3 nm)/CrRu (30 nm)/glass. The third intermediate layer is TiN (2 nm)- ZrN (20 vol.%, 40 vol.%)/TiN (3 nm)/CrRu (30 nm)/glass. All the FePt layers were fabricated by dc-sputtering of a Fe_55_Pt_45_ alloy target with Ar working pressure of 10 mTorr. The deposition temperature of FePt, ZrN and TiN were fixed at 400 °C, and the deposition temperature of CrRu was 280 °C. The sputtering rate of FePt target, ZrN target, TiN target and CrRu target were 0.08 nm/s, 0.11 nm/s, 0.02 nm/s and 0.2 nm/s, respectively. The FePt-SiO_2_-C films would be obtained by co-sputtering the FePt target, SiO_2_ target and C target. TiN-ZrN films were obtained by co-sputtering the TiN and ZrN target. At the end of sputtering, the samples were left to cool to room temperature in the main sputtering chamber and after which, taken out for characterization.

### Characterization of FePt films

The crystallographic texture was examined with X-ray diffraction (XRD) using Cu K_α_ radiation. The microstructures of the films were characterized by JEOL 2010F transmission electron microscopy (TEM). The morphologies of the samples were examined by Zeiss Supra 40 FE scanning electron microscopy (SEM). The magnetic properties were measured using the alternating gradient force magnetometer (AGFM) at a maximum applied field of 20 kOe at room temperature and using the superconducting quantum interference device (SQUID) at a maximum applied field of 60 kOe at room temperature.

## Additional Information

**How to cite this article**: Dong, K. F. *et al.* Columnar structured FePt films epitaxially grown on large lattice mismatched intermediate layer. *Sci. Rep.* **6**, 34637; doi: 10.1038/srep34637 (2016).
